# Research on Density Prediction of Laser Powder Bed Fusion Process Parameters for IN718 Nickel-Based Superalloy Based on Machine Learning

**DOI:** 10.3390/ma19122455

**Published:** 2026-06-08

**Authors:** Lina Zhu, Jifeng Wang, Zongxian Song, Hongye Guo, Bohan Li, Yong Liu

**Affiliations:** 1 Wheel Rail Center, Tianjin Research Institute for Advanced Equipment, Tsinghua University, Tianjin 300300, China; 2 School of Aeronautics and Astronautics, Sino-German University of Applied Sciences, Tianjin 300350, China; 3 School of Mechanical Engineering, Sino-German University of Applied Sciences, Tianjin 300350, China; 4 School of Materials Science and Engineering, Tianjin University, Tianjin 300350, China

**Keywords:** selective laser melting (SLM), IN718 nickel-based superalloy, machine learning, data augmentation, RBF interpolation, generative adversarial network (GAN), density prediction, support vector regression (SVR)

## Abstract

This study addresses the challenge of modeling the complex non-linear relationship between process parameters and relative density in selective laser melting (SLM) of IN718 nickel-based superalloy under small-sample conditions. A data-driven prediction framework integrating data augmentation, physics-informed feature engineering, machine learning, and model interpretability analysis was developed and systematically validated. Fourteen sets of experimental data covering both vertical and horizontal building directions were collected by varying laser power (P), scan speed (v), and hatch spacing (h). To overcome the small-sample limitation, three augmentation strategies—radial basis function (RBF) interpolation, generative adversarial network (GAN), and K-nearest neighbors (KNN)—were systematically compared under unified physical constraints combining local perturbation and volumetric energy density (E_vol) filtering, with Pearson correlation coefficient consistency used to select the optimal strategy. Eight physically meaningful input features were constructed, including E_vol and line energy density (E_line), explicitly embedding SLM process physics into the learning framework. Support vector regression (SVR), random forest (RF), and artificial neural network (ANN) models were trained and their hyperparameters were systematically optimized via exhaustive grid search combined with leave-one-out cross-validation (LOO-CV), ensuring robust model selection under small-sample constraints. A physics-based baseline model (E_vol quadratic fitting, LOO-CV average R^2^ = 0.2534) was established to quantify the gain of machine learning over empirical formulas. LOO-CV results show that ANN achieves the highest average R^2^ of 0.9269, followed by SVR (0.9148) and RF (0.8393), all of which substantially outperform the physical baseline. Feature importance analysis reveals that E_vol accounts for 51.58% of the predictive power, and ablation experiments confirm that introducing physics-derived features improves the average R^2^ by 0.0246 compared with raw process parameters alone. To further elucidate the predictive mechanism of the optimal ANN model, Partial Dependence Plot (PDP) analysis was conducted for all eight input features, visualizing their marginal effects on predicted density and confirming physical consistency with SLM mechanisms. This framework provides a reliable, interpretable, data-driven solution for intelligent SLM process optimization with limited experimental data.

## 1. Introduction

Selective laser melting (SLM), a representative powder bed fusion additive manufacturing technology, enables the near-net-shape fabrication of geometrically complex components and has been widely adopted in the aerospace industry [1,2]. IN718 nickel-based superalloy, accounting for more than 40% of aero-engine hot-section components [1], is among the most frequently processed materials via SLM. Relative density is a critical quality indicator in SLM-fabricated parts, as internal porosity, cracks, and lack-of-fusion defects significantly degrade mechanical properties and fatigue life [3,4].

However, SLM densification is governed by a highly non-linear laser–powder thermal interaction process. Laser power (P), scan speed (v), and hatch spacing (h) exhibit strong coupling effects on melt pool dynamics, solidification behavior, and defect formation [4,5,6,7]. Conventional optimization approaches, such as orthogonal experiments and response surface methodology (RSM), are cost-intensive and time-consuming and struggle to capture the multi-physics coupling inherent in SLM. Machine learning (ML) has demonstrated substantial potential for predicting materials properties [8,9]; however, the high experimental cost of SLM trials often yields datasets with fewer than 20 samples, making direct ML modeling prone to severe overfitting [8].

To address the small-sample challenge, data augmentation has been adopted in adjacent fields. GAN-based and VAE-based generative strategies have been applied to fatigue-life datasets for metallic materials [10,11,12], and physics-constrained augmentation coupled with SMOTE has shown promise in structural fatigue applications [9,13]. In the context of SLM-fabricated nickel-based superalloys, recent work has attempted to predict internal densification directly from surface features using machine learning [14,15], demonstrating the feasibility of data-driven approaches for this material system. Nevertheless, several critical gaps remain in SLM density prediction. First, no systematic comparison of multiple augmentation strategies under unified physical constraints has been conducted for SLM small-sample problems. Second, existing ML studies in SLM largely treat the process–density relationship as a generic regression problem using raw process parameters (P, v, h), without explicitly embedding physical mechanisms into the learning process, which limits model interpretability and extrapolation capability [8]. Third, although SVR and RF have demonstrated competitive predictive performance for LPBF process–property relationships under limited experimental data [15,16], and comparative studies indicate that model performance rankings are highly task-dependent across different material systems and loading conditions [16], the justification for model complexity is rarely established through ablation studies or comparison with simple physics-based baselines, making it difficult to assess whether machine learning genuinely outperforms established engineering formulas.

The present work addresses these gaps through the following original contributions: (1) Three augmentation strategies—RBF interpolation, GAN, and KNN—are systematically compared under unified physical constraints (local perturbation + E_vol filtering) using three quantitative evaluation metrics, providing an evidence-based selection of the optimal strategy rather than an ad hoc choice. (2) Eight physics-informed features are constructed and their contributions quantified via ablation experiments and feature importance analysis, explicitly linking model inputs to SLM densification physics. (3) A physics-based baseline model (E_vol quadratic fitting) is introduced under the same LOO-CV framework to quantify the performance gain of ML over traditional empirical formulas and to justify model complexity. (4) Hyperparameters of all three machine learning models (SVR, RF, and ANN) are systematically optimized through exhaustive grid search combined with LOO-CV, enabling reliable model selection and mitigating overfitting risks inherent in small-sample scenarios. (5) Partial Dependence Plot (PDP) analysis is applied to the optimal ANN model to visualize the marginal effect of each input feature on the predicted density, providing physical interpretability that goes beyond standard accuracy metrics and confirming consistency with known SLM mechanisms. Together, these contributions establish a complete, physically interpretable, and experimentally validated prediction framework for SLM density under small-sample conditions.

## 2. Materials and Methods

### 2.1. IN718 Nickel-Based Superalloy Powder

The nickel-based superalloy powder used in this study was IN718 powder supplied by Jiangsu Vilory New Materials Co., Ltd., Xuzhou, Jiangsu, China, with a particle size distribution of 15–53 μm. The chemical composition of the powder is presented in Table 1.

### 2.2. SLM Forming of IN718 Nickel-Based Superalloy

**Figure 1:**
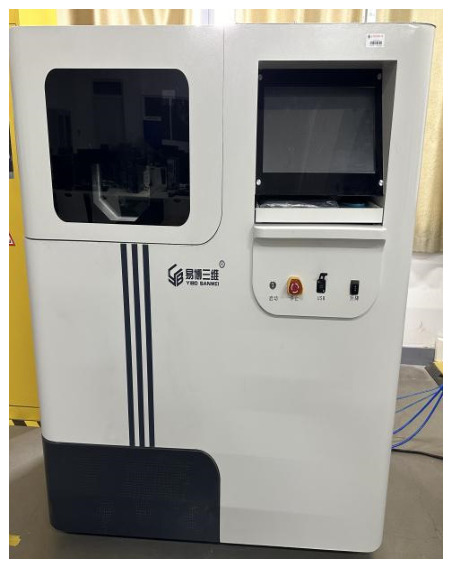
Yibo 3D IGAM-1 Printer.

The experiments were conducted using the Yibo 3D IGAM-1 printer (Beijing Yibo 3D Technology Co., Ltd., Beijing, China) (Figure 1), which consists of a high-reliability computer, various reliable control modules, motor drive units, and various sensors. The main unit comprises five basic components: the working cylinder, the powder spreading device, the powder feeding device, the electrical circuit system, and the machine body and casing, which together perform the system’s processing and transmission functions. A continuously adjustable constant-temperature water cooling unit and external piping are used to cool the laser, improve laser energy stability, protect the laser, and extend its service life.

The maximum forming space of this printer is 150 mm × 150 mm × 128 mm, with a forming accuracy of 100 mm ± 0.1 mm. The laser uses a high-performance optical fiber with a 500 W continuous power; the maximum scanning speed is 5000 mm/s, and the maximum processing speed is 1000 mm/s. It can form iron-, nickel-, and copper-based alloys, as well as titanium alloys, stainless steel, and high-temperature alloys.

**Figure 2:**
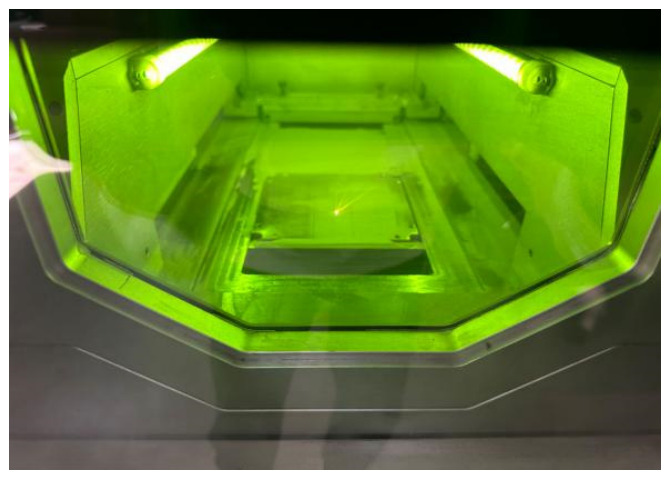
Laser Scanning Process.

**Figure 3:**
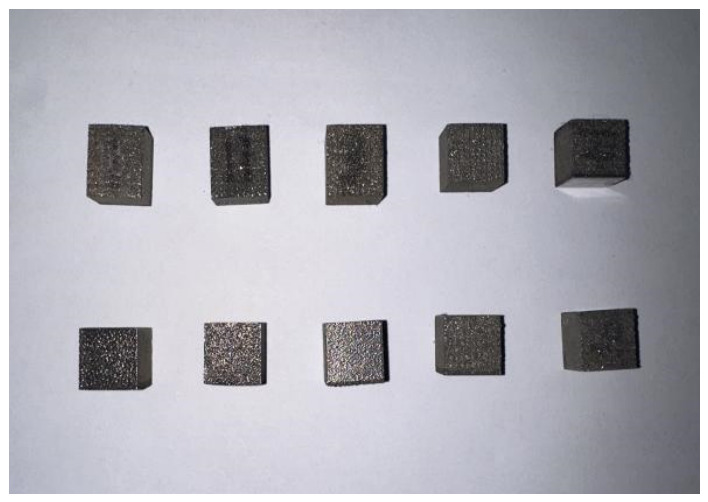
As-Printed Specimens.

The printing procedure comprises four main stages: (1) preparation—the substrate was ground and cleaned with acetone, and the IN718 powder was dried at 200 °C for 2 h to remove moisture; (2) system start-up—the water cooler was switched on 15 min in advance to stabilize the water temperature at 22 °C, after which the main computer, powder cylinder, and spreading system were initialized and the optical path was verified; (3) parameter setting—the target laser power, scanning speed, and hatch spacing were entered, and the build chamber was purged with protective argon gas until the oxygen content was negligible; and (4) printing—the laser selectively melted successive powder layers following a preset scan path (Figure 2), with the substrate lowered by one layer thickness after each layer and fresh powder uniformly spread until the part was fully fabricated (Figure 3).

**Figure 4:**
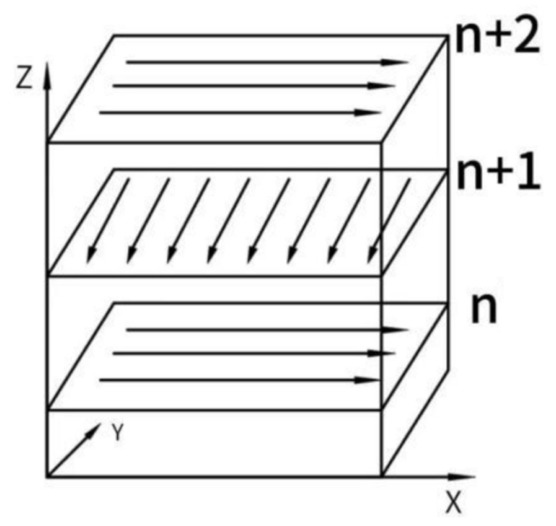
Scanning Strategy.(The arrow indicates the scanning direction.)

A cross-scanning strategy was adopted in this experiment, i.e., the scanning direction of each layer is rotated 90° relative to the previous layer, and layers n and n + 2 share the same scanning direction (Figure 4). All specimens were used in the as-printed state without hot isostatic pressing or heat treatment.

### 2.3. Density Measurement

**Figure 5:**
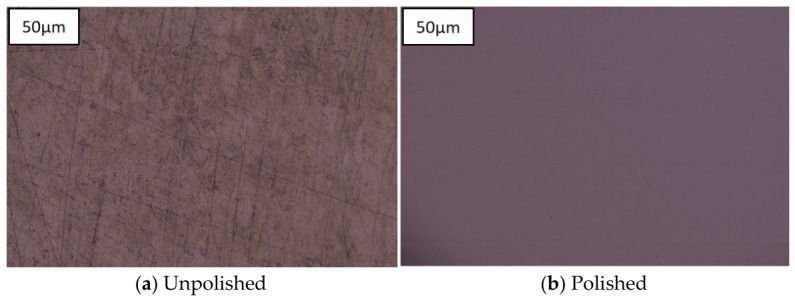
Comparison of Metallographic Micrographs Before and After Polishing.

The cut surfaces were ground sequentially using 240#, 400#, 800#, 1000#, 1500#, and 2000# abrasive papers and then polished with a polishing cloth. As summarized in Figure 5, the unpolished surface exhibited pronounced scratches and surface contaminants that obscured the intrinsic pore morphology, whereas the polished surface displayed a mirror-like finish with clearly discernible pore boundaries. This contrast confirms that the grinding and polishing protocol effectively eliminates surface artifacts, ensuring reliable subsequent porosity quantification.

**Figure 6:**
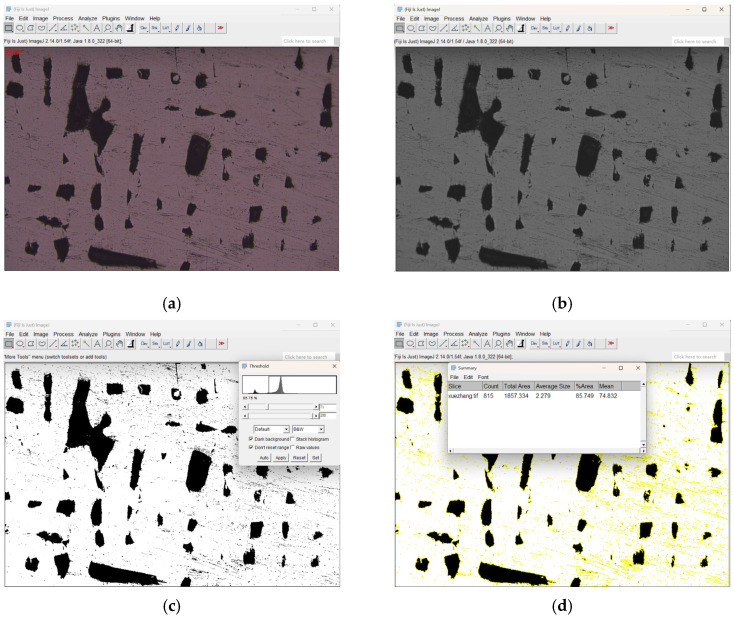
ImageJ Porosity Analysis Procedure for Density Measurement. (**a**) Original Image; (**b**) Background Adjustment; (**c**) Color Adjustment Figure; (**d**) Density Measurement Results.

Porosity was evaluated using ImageJ 1.54r software through a standardized four-step image processing workflow, as summarized in Figure 6. First, the original metallographic image (Figure 6a) was converted to 8-bit grayscale to eliminate color variations and enhance contrast (Figure 6b). An automatic global threshold was then applied to segment the image into a binary representation, where pores appeared as black features against a white metallic matrix (Figure 6c). Finally, the Analyze Particles function was used to compute the total area fraction of black pixels, yielding the porosity percentage. The density of each specimen was subsequently derived as (100% − porosity). As illustrated in Figure 6d, the binary image clearly delineates individual pores with well-defined boundaries, confirming that the selected threshold adequately distinguishes true porosity from surface artifacts. This fully automated procedure ensures objective and reproducible evaluation across all specimens, providing the vertical and horizontal density values reported in Table 2.

## 3. Raw Data Construction and Processing

### 3.1. Raw Data Construction

In the experiments, the Yibo 3D IGAM-1 printer was used to print specimens by adjusting three parameters: laser power (P), scanning speed (v), and hatch spacing (h). The effects of these parameters on the Density and microhardness of the specimens were further analyzed. To ensure experimental rigor and reproducibility, we performed four replicate prints for each set of parameters and calculated the average value from the four trials. Furthermore, considering the high sensitivity of the LPBF process to machine-specific factors, the following variables were held constant throughout the experiment: a laser spot size of 0.05 mm, a powder particle size range of 15–53 μm, and a shielding gas flow rate adjusted to maintain the chamber oxygen content below 1%. For specimen identification and specific printing parameters, please refer to Table 2 and Table 3.

The predictive scope of this model is limited to the experimental parameter space investigated in this study (P: 200–335 W, v: 600–1200 mm/s, h: 0.06–0.12 mm). Since the experimental design focused on establishing a process window for high relative density, the model’s predictions in extreme defect regimes, such as keyhole or lack-of-fusion, require further validation.

### 3.2. Data Augmentation

Since the raw dataset contains only 14 samples, this volume is insufficient for machine learning workflows and may lead to excessively large prediction errors. Therefore, a data augmentation step was incorporated prior to the machine learning pipeline to expand the dataset. Due to the limited dataset, we employed three distinct data augmentation methods—RBF, GAN, and KNN—to process the raw data. RBF was selected for its inherent suitability for continuous variables, which aligns well with the continuous nature of SLM process parameters. As a representative of mainstream deep learning, GAN is capable of capturing more complex data patterns compared to traditional interpolation methods and has served as a benchmark in most existing literature. To avoid unnecessary complexity, we utilized the KNN model as a baseline. Given its training-free nature and minimal computational cost, the KNN model serves as a reference to verify whether the more sophisticated methods (RBF and GAN) provide substantial performance gains. The results and descriptions of the three augmentation methods are presented below [17].

#### 3.2.1. Radial Basis Function (RBF) Interpolation

This method employs RBF thin-plate spline interpolation to construct a continuous density prediction surface in the physical parameter space. Thin-plate spline interpolation is used to construct a smooth surface f(x, y) from a set of discrete points (xᵢ, yᵢ) with corresponding function values zᵢ, such that the surface passes through all known points while minimizing the overall bending energy. The objective function can be written as (1):

(1) $$E_{d}=\iint_{R^{2}}\left[{\left(\frac{{𝜕}^{2}f}{𝜕x^{2}}\right)}^{2}+2{\left(\frac{{𝜕}^{2}f}{𝜕x𝜕y}\right)}^{2}+{\left(\frac{{𝜕}^{2}f}{𝜕y^{2}}\right)}^{2}\right]dx\;dy$$

Subject to the constraint:

f(x_i, y_i) = z_i, i = 1, 2, …, N

where N is the number of known experimental points; $E_{d}$ is the total bending energy of the surface. The terms

$${\left(\frac{{𝜕}^{2}f}{𝜕x^{2}}\right)}^{2}and\;{\left(\frac{{𝜕}^{2}f}{𝜕y^{2}}\right)}^{2}$$

represent the squared second-order derivatives (curvatures) of the surface in the x and y directions, respectively, indicating the degree of bending along those directions. The term

$$2{\left(\frac{{𝜕}^{2}f}{𝜕x𝜕y}\right)}^{2}$$

is the square of the mixed partial derivative, representing the bending energy due to surface torsion. The coefficient 2 is included for consistency with the thin-plate strain energy expression in elasticity theory.

The interpolation constraint *f*(*x_i*, *y_i*) = *z_i* requires the constructed surface to pass exactly through every known experimental point.

**Figure 7:**
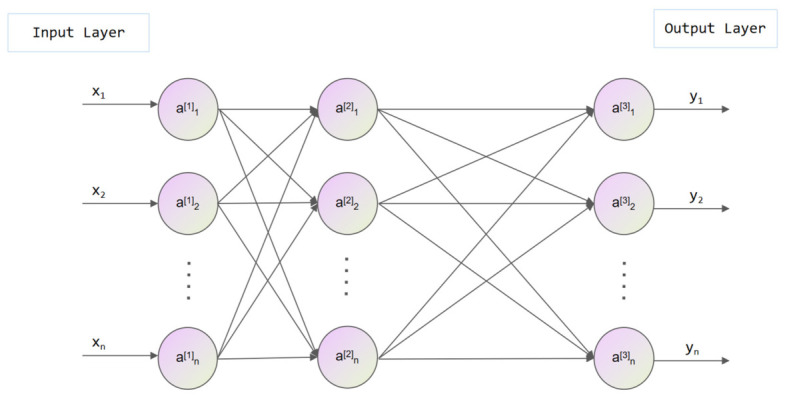
Network Architecture of the Radial Basis Function (RBF).

The specific network structure is shown in Figure 7.

The only way to influence the prediction results of an RBF network is to adjust the parameterized shape of the radial basis function for each input neuron. The higher the value of the spread parameter sp, the flatter the radial basis function becomes [18]. Therefore, optimizing the sp parameter involves performing multiple trials to identify the value that yields the best prediction accuracy.

Due to the high cost of experiments, collecting large amounts of measured data is prohibitively expensive. To systematically optimize the sp parameter, model prediction accuracy is evaluated using Leave-One-Out Cross-Validation (LOO-CV). For each candidate sp value, the training and test set RMSEs are calculated, normalized, and combined into an equal-weight compromise score:

Compromise score = 0.5 × norm(Train RMSE) + 0.5 × norm(Test RMSE)

**Figure 8:**
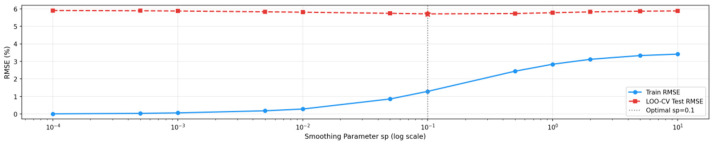
Optimization Results.

The sp value that yields the lowest compromise score is considered optimal. The evaluation metric used is the Root Mean Square Error (RMSE):

$$RMSE=\sqrt{\frac{1}{n}\sum_{i=1}^{n}(y_{i}-{\hat{y}}_{i}{)}^{2}}$$

where $y_{i}$ is the actual value, ${\hat{y}}_{i}$ is the predicted value, and n is the number of data points. The specific optimization data are shown in Figure 8.

#### 3.2.2. Generative Adversarial Network (GAN)

A GAN consists of two main components: the generator and the discriminator.

Generator: The generator can be any neural network architecture. It takes random noise as input and produces generated samples as output. The generator’s objective is to produce samples as close as possible to the distribution of real samples to deceive the discriminator.

Discriminator: The discriminator can similarly be a neural network of any architecture. It takes either real or generator-generated samples as input and outputs a probability indicating the likelihood that the input is a real sample. The discriminator’s objective is to distinguish as accurately as possible between real and generated samples.

**Figure 9:**
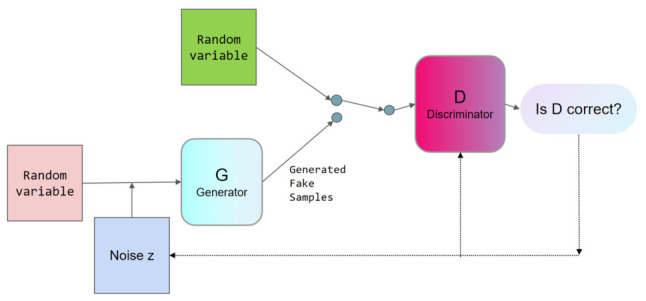
Architecture of the Generative Adversarial Network (GAN).

During training, the two networks compete and optimize each other to increasingly approximate the true data distribution. The training structure is illustrated in Figure 9.

The objective function of the novel GAN proposed in this paper for synthetic data generation is shown in Equation (2) [19]:

(2) $$\underset{G}{\min}\underset{D}{\max}V(D,G)=E_{X∼P\;data(x)}[\log D(x)]+E_{Z∼P_{z}(z)}[\log(1-D(G(z)))]$$

where G represents the generator parameters, whose goal is to generate samples similar to real data; D represents the discriminator parameters. The term

$$E_{X∼P\;data(x)}[\log D(x)]$$

denotes the expectation over samples x drawn from the real data distribution p_data(x), computed as the logarithm of the discriminator’s predicted probability for these real samples. The term

$$E_{Z∼P_{z}(z)}[\log(1-D(G(z)))]$$

represents the generator’s input distribution, where p_z(z) denotes the noise distribution sampled in the generator; the noise is fed into the generator to produce a sample, which is then passed to the discriminator. The generator aims to maximize this term, making it increasingly difficult for the discriminator to distinguish generated samples from real ones.

#### 3.2.3. KNN (K-Nearest Neighbors)

K-Nearest Neighbors (KNN) is a non-parametric, lazy learning supervised algorithm applicable to both classification and regression tasks. The fundamental principle of KNN is to identify the K-nearest neighbors of a new sample in the training dataset and predict its target category or numerical value based on those neighbors. A critical aspect of this approach is selecting an appropriate distance metric to quantify sample proximity. In this study, we focus on the most common metric: Euclidean distance.

(3) $$d(x,y)=\sqrt{\sum_{i=1}^{n}{\left(x_{i}-y_{i}\right)}^{2}}$$

**Figure 10:**
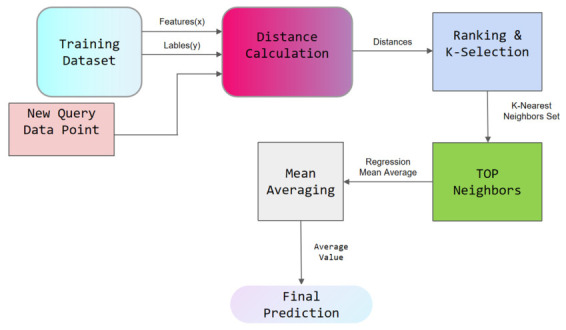
Architecture of the KNN.

For a new sample x, the KNN predicted value $\hat{y}$ is

(4) $$\hat{y}=\frac{1}{K}\sum_{i=1}^{K}y_{\left(i\right)}$$

where $y_{\left(i\right)}$ denotes the true values of the K-nearest neighbors. The training structure is illustrated in Figure 10.

### 3.3. Optimization of Data Augmentation Based on Physical Constraints and Local Perturbation

Using the initial model for data augmentation may yield parameter combinations that cause extremely high or low volumetric energy densities (E_vol). These moves generated samples outside the valid range and far from the original data, leading to uncontrolled extrapolation and uncertain physical reliability.

To address this, this study employs two combined strategies to optimize the data augmentation process.

Local perturbation: New samples are generated within a ±10% parameter range around each original experimental point, ensuring that the data remains within a reliable domain.

E_vol Physical Constraint Filtering: Global random sampling is restricted to within ±15% of the E_vol range derived from the original dataset to exclude physically implausible parameter combinations. Here, t = 0.05 mm represents the powder layer thickness, which was held constant across all experimental groups in this study.

The E_vol values for the 14 original experimental points range from 37.5 to 114.58 J/mm^3^.

(5) $$E\_vol=\frac{P}{\left(v\cdot h\cdot t\right)}$$

The combined application of these two strategies ensures the physical reliability of the augmented data while maintaining the diversity of the parameter space.

### 3.4. Data Correlation Analysis

The three methods described above were used to augment the raw data, respectively. Before training the machine learning model, it is necessary to evaluate the consistency and reliability of the expanded data relative to the original data. A widely adopted method for determining the correlation among a set of variables is the Pearson Correlation Coefficient (PCC), as given in Equation (6):

(6) $$P=\frac{\sum \left(X_{i}-\bar{X}\right)(Y_{i}-\bar{Y})}{\sqrt{\sum {\left(X_{i}-\bar{X}\right)}^{2}\sum {\left(Y_{i}-\bar{Y}\right)}^{2}}}$$

where Xᵢ and Yᵢ are the values of two feature variables, and $\bar{X}$ and $\bar{Y}$ are their respective means.

When the raw process parameters (P, v, and h) are used as independent input variables, the model is required to implicitly learn the non-linear coupling relationships between them from a limited dataset, which results in inefficient learning in small-sample scenarios. To address this, the feature space was expanded into an 8-dimensional physics-inspired input space, and its correlations were analyzed (Table 4) [20,21].

**Figure 11:**
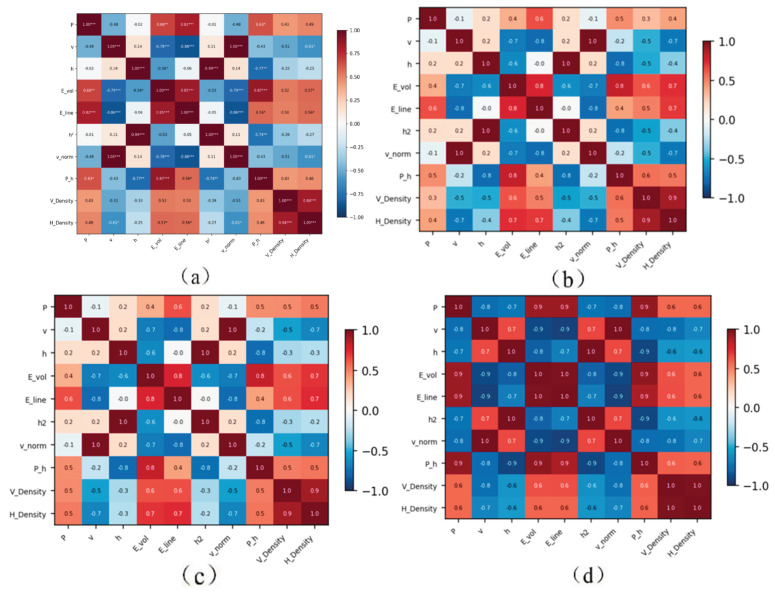
Pearson Correlation Coefficient Heatmaps of the Original and Augmented Datasets. (**a**) Original Dataset; (**b**) RBF; (**c**) KNN; (**d**) GAN. (Asterisks indicate the statistical significance of the correlations.)

PCC heatmaps were obtained for the different density-related parameters across the original dataset and the three augmented datasets, as shown in Figure 11. It can be observed that the RBF-interpolated augmented data exhibits the closest correlation structure to the original data. This confirms the reliability of the RBF interpolation augmentation strategy. Therefore, in this work, the three augmentation approaches are first distinguished, and their applications are compared to provide a more comprehensive perspective for describing the Density of IN718 nickel-based alloy printed parts. Overall, using the RBF interpolation-augmented dataset for subsequent ML model construction is well justified.

The Pearson correlation map of the original data reveals a positive correlation between relative density (Density) and E_vol, with a correlation coefficient of 0.55, indicating that, under the influence of factors such as energy density, an increase in E_vol significantly promotes an increase in density. The correlation between P and Density is 0.46, indicating a positive relationship: an increase in P improves the final density. The correlation between v and Density is −0.56, indicating a significant negative relationship: an increase in v has a notable inhibitory effect on density. The correlation between h and Density is −0.29, and the correlation between E_line and Density is 0.53.

By comparing the Pearson correlation coefficient map of the original data with that of the RBF-augmented data, we can observe the correlation between the parameter h and Density: in the original data, the correlation between h and Density is −0.29, whereas in the M1: RBF-augmented dataset, it shifts to −0.45. This implies that, in data augmented by the RBF model, the negative impact of the h parameter (hatch spacing) on density is significantly amplified.

## 4. Machine Learning Model Construction and Optimization

### 4.1. Data Preprocessing

Data preprocessing is a critical step in machine learning, aimed at transforming raw data into a standardized form suitable for model training. The primary reason is that parameters such as laser scanning power P, scanning speed v, and hatch spacing h have different units and dimensions. Unprocessed data may contain errors or biases that degrade model performance, whereas appropriate preprocessing can significantly improve model accuracy, robustness, and generalization, thereby laying a reliable foundation for subsequent modeling.

#### 4.1.1. Data Normalization

After data augmentation, an eight-dimensional variable set was obtained, comprising laser scanning power P, scanning speed v, hatch spacing h, vertical Density, horizontal Density, volumetric energy density E, linear energy density E_line, squared hatch spacing h^2^, normalized speed v_norm, and power-to-spacing ratio P_h. Since these variables have different scales and units, they impede uniformity and may lead to longer training times and convergence challenges. Therefore, it is essential to preprocess the dataset prior to using it for ML model training. This process is intended to reduce the dimensional complexity and redundancy in the data. The normalization Equation (7) is used for this purpose:

(7) $$X_{norm}=\frac{X-X_{min}}{X_{max}-X_{min}}$$

where X_norm, X, X_min, and X_max denote the normalized value, original value, minimum value, and maximum value of variable X, respectively.

#### 4.1.2. Dataset Partitioning

It is essential to ensure that the established machine learning model is both reliable and well-generalized. The augmented dataset is split into a training set and a validation set in a 7:3 ratio, while the original data serves as the test set and is excluded from the training process. The training set is used to train a density prediction model iteratively. In contrast, the validation set facilitates hyperparameter optimization and selection of the optimal hyperparameters. In addition, Leave-One-Out Cross-Validation (LOO-CV) is applied to the training data to mitigate the influence of data randomness on prediction results. Finally, the original data is fed into the trained model for prediction, thereby evaluating the model’s predictive performance.

### 4.2. Machine Learning Model Construction

To address the small-sample non-linear regression problem between SLM process parameters and relative density, three representative machine learning models were selected using a systematic methodological approach. Support vector regression (SVR) is well-suited to small-sample scenarios, as its decision boundary is defined by only a subset of support vectors rather than the entire training dataset, thereby conferring strong generalization under limited data conditions. The RBF kernel was adopted to enable implicit high-dimensional non-linear mapping, consistent with the complex coupling between process parameters and density. Random forest (RF), as an ensemble learning method, reduces overfitting risk through the averaging of multiple decision trees and exhibits robust performance under small-sample conditions; its built-in feature importance output further supports the interpretability analysis conducted in this study. An artificial neural network (ANN) was included as a representative deep learning approach to establish an upper-bound reference for predictive accuracy and to assess whether data volume constitutes a performance bottleneck. The three model classes represent kernel methods, ensemble methods, and neural network methods, respectively, providing systematic and representative coverage of mainstream small-sample regression strategies.

**Figure 12:**
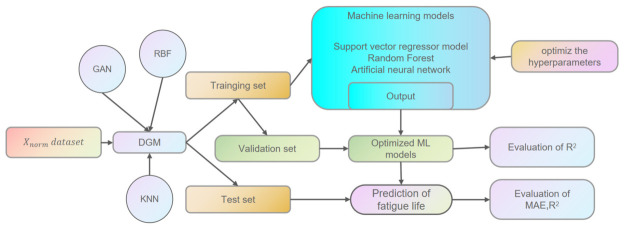
Flowchart of the Machine Learning Model.

After data augmentation and preprocessing, the 8-dimensional physical features were used as input variables to train the machine learning model on the training set. After hyperparameter optimization, the optimal machine learning model is obtained, and the R^2^ value on the validation set within the training set is calculated to assess the fit of each ML model. The optimal ML model is then used for density prediction, with R^2^ and MAE as evaluation metrics to assess its generalization and accuracy. The described process is illustrated in Figure 12.

To assess the rationale for the complexity of the selected machine learning models, a quadratic polynomial fit of the volumetric energy density (E_vol) is introduced as a physical baseline. Adopting the same Leave-One-Out Cross-Validation (LOO-CV) framework as used for the ML models, we compare the prediction accuracy of the physical baseline with that of the ML models to quantitatively evaluate the practical gains of the machine learning approach over traditional physical empirical formulas and to justify the necessity of the increased model complexity.

#### 4.2.1. Support Vector Regression (SVR)

**Figure 13:**
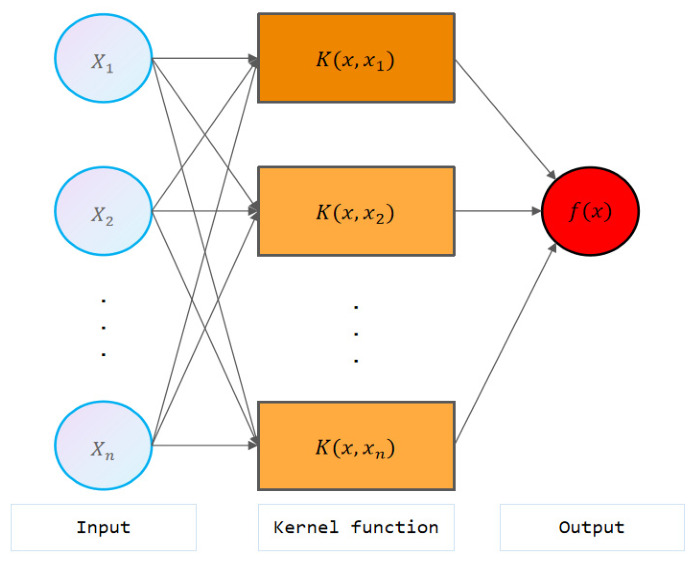
Architecture of the Support Vector Regression (SVR) Model.

After data augmentation and preprocessing, the data is fed into Support Vector Regression (SVR) for training. The objective is to identify an optimal hyperplane that minimizes the model’s prediction error on the training data while tolerating a specified level of error. The training involves an optimization problem in which the penalty parameter (C), kernel function parameter (γ), and tolerance (ε) are fine-tuned using methods such as Leave-One-Out cross-validation. This stage aims to evaluate model performance on validation data and select the optimal hyperparameter configuration to prevent underfitting or overfitting. Available kernel functions include the linear kernel, radial basis function (RBF), polynomial kernel (poly), and sigmoid. The regression function is expressed in Equation (8) [22,23]:

(8) $$f(x)=\sum_{i=1}^{n}(\alpha_{i}-\alpha_{i}^{*})\cdot K(x_{i},x_{j})+b,\alpha_{i},\alpha_{i}^{*}\in \left[0,C\right]$$

where $\alpha_{i}$ and $\alpha_{i}^{*}$ are Lagrange multipliers, b is the bias term, and C is the penalty parameter. K(xᵢ, xⱼ) denotes the kernel function with radial basis (RBF), linear, and polynomial kernel distributions. The SVR structure is shown in Figure 13, where the eight-dimensional features described above are used as the input parameters x, and Density serves as the output dependent variable.

#### 4.2.2. Random Forest (RF)

The training stage of the random forest regression model involves constructing multiple uncorrelated decision trees. In this study, the eight-dimensional feature parameters serve as the input parameters, with Density as the output dependent variable y_RF. The training dataset is divided into k sub-datasets, each used to train a different decision tree. For each decision tree, a predicted output vector is computed. Finally, the result is obtained by averaging the predicted output vectors from all decision trees, as given by Equation (9) [23,24]:

(9) $$y_{RF}=\frac{1}{k}\sum_{p=1}^{k}y_{p}^{pre}$$

**Figure 14:**
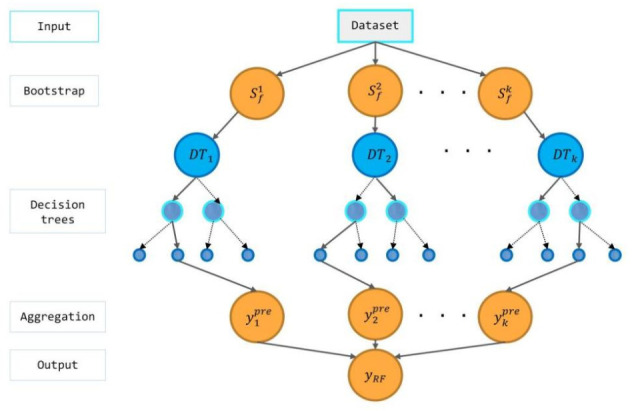
Architecture of the Random Forest (RF) Model.

Four important parameters in the random forest model include n_estimators, min_samples_split, min_samples_leaf, and max_depth. Grid hyperparameter optimization is used in this study to tune random forest parameters. The random forest structure is shown in Figure 14.

#### 4.2.3. Artificial Neural Network (ANN)

Artificial Neural Networks (ANNs) consist of numerous neurons, including an input layer associated with the prediction target. The input layer receives input data, with each node representing an input feature, along with hidden layers. The hidden layers facilitate learning of complex data relationships. There may be multiple hidden layers, each comprising many nodes. The output layer produces the prediction results, with each node corresponding to an output class or value. Neurons are the basic units of ANNs. Input data passes through the input layer and propagates sequentially through the hidden layers, ultimately producing results in the output layer. Each node in the hidden layers receives input values from the previous layer, which are then processed by weights, bias functions, and activation functions to generate outputs that are transmitted to the next layer. The regression function of the i-th neuron yᵢ is expressed by Equation (10) [25]:

(10) $$y_{i}=f_{i}\left(\sum \omega_{i,j}x_{j}-t_{i}\right)$$

where $\omega_{i,j}$ is the weight of the neural network, $x_{j}$ is the input to the current neuron node, $f_{i}$ is the activation function, and $t_{i}$ is the threshold parameter.

**Figure 15:**
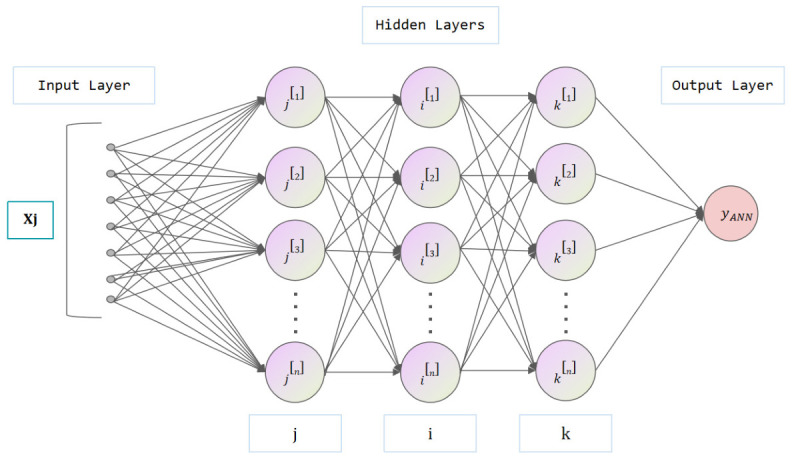
Architecture of the Artificial Neural Network (ANN) Model.

The core idea is to use the training dataset to continuously adjust the weights via backpropagation until the model accurately predicts the input data. Commonly used optimization algorithms include gradient descent and stochastic gradient descent, which update the weights at each iteration to minimize the error function. The error function is given by Equation (11) [26,27]:

(11) $$E=\frac{1}{2}\sum_{i=1}^{n}\left\|y_{i}-y_{i}^{true}\right\|$$

where E is the error function, n is the number of samples in the dataset, $y_{i}^{true}$ is the actual output of the regression function, and $y_{i}$ is the true density value from the density experiment. This study employs a Multi-Layer Perceptron (MLP) ANN regressor for modeling. The ANN model structure is shown in Figure 15.

#### 4.2.4. Physical Baseline Model (Quadratic Fitting of E_vol)

Based on the SLM-forming mechanism, relative density exhibits a non-linear relationship with E_vol; therefore, a quadratic polynomial is selected as the fitting function:

(12) $$\rho =a\cdot E_{vol}^{2}+b\cdot E_{vol}+c$$

where ρ denotes the predicted density, and a, b, and c are the coefficients to be fitted, which are determined by minimizing the sum of squared residuals:

(13) $$\underset{a,b,c}{\min}\sum_{i=1}^{n}{\left(\rho_{i}-a\cdot E_{vol,i}^{2}-b\cdot E_{vol,i}-c\right)}^{2}$$

The volumetric energy density is calculated by the following equation:

(14) $$E_{vol}=\frac{P}{v\cdot h\cdot t}$$

### 4.3. Model Evaluation

To evaluate the accuracy of the machine learning models in predicting Density, this study uses the coefficient of determination (R^2^) and Mean Absolute Error (MAE) as evaluation metrics. Among these, R^2^ accurately reflects the regression model’s fit; the closer its value is to 1, the higher the model’s accuracy and the better its fit. MAE represents the mean absolute distance between predicted and actual values; the closer it is to 0, the better the model’s predictive capability. The calculation formulas for R^2^ and MAE are given in Equations (15) and (16), respectively:

(15) $$R^{2}(y,y^{pre})=1-\frac{\sum_{i=1}^{n}{\left(y_{i}^{true}-y_{i}^{pre}\right)}^{2}}{\sum_{i=1}^{n}{\left(y_{i}^{true}-y_{mean}\right)}^{2}}$$

(16) $$\mathrm{MAE}(y,y^{\mathrm{pre}})=\frac{1}{n}\sum_{i=1}^{n}\left|y_{i}^{\mathrm{true}}-y_{i}^{\mathrm{pre}}\right|$$

where $y_{i}^{true}$ denotes the i-th original data value, $y_{i}^{pre}$ denotes the i-th predicted value, and $y_{mean}$ denotes the mean value of the original data.

### 4.4. Hyperparameter Optimization

During machine learning model training, model performance and generalization are improved by adjusting hyperparameters. Hyperparameters are parameters preset before model training whose values cannot be learned automatically during training and must be manually adjusted. The selection of hyperparameters is critical to model performance and generalization capability. To accurately evaluate model performance across different hyperparameter settings, Leave-One-Out cross-validation is typically employed: the training data is split into a training set and a validation set; the training set is used for iterative model training, and the validation set is used to assess the model’s performance. Before initiating the optimization process, the hyperparameter and parameter value ranges must be defined, and an appropriate hyperparameter optimization method must be selected, such as grid search, random search, or Bayesian optimization. The optimization parameter sets for each ML model are detailed in Table 5.

For the SVR model, three hyperparameters were optimized: the penalty parameter C with a range of C ∈ [10, 20, …, 140, 150] (15 equidistant values); the tolerance parameter ε with values ε ∈ [0.001, 0.01, 0.1, 1, 10] (5 exponentially spaced values); and the kernel coefficient γ with values γ ∈ [0.001, 0.01, 0.1, 1, 10] (5 exponentially spaced values). This parameter configuration systematically explores the effect of different hyperparameter combinations on model performance.

For the RF model, four hyperparameters were optimized: the total number of decision trees n_estimators ∈ [50, 100, 150, 200, 500]; the minimum number of samples required to split an internal node min_samples_split ∈ [2, 4, 6, 8, 10, 16]; the minimum number of samples required at a leaf node min_samples_leaf ∈ [1, 2, 4]; and the maximum tree depth max_depth ∈ [None, 10, 20, 30, 40, 50], where None indicates no depth limit.

For the ANN model, four key hyperparameters were optimized. The hidden layer structure (hidden_layer_sizes) defines the network depth and number of neurons as a list of tuples; for example, (20) represents a single hidden layer with 20 neurons. The specific ranges are: first layer neuron count i ∈ [20, 30, …, 70, 80]; subsequent layer counts j ∈ [10, 20, 40] and k ∈ [10, 15, 20]. The activation function (activation) options include the linear function ‘identity’ (17), rectified linear unit ‘RELU’ (18), sigmoid function (19), and hyperbolic tangent function ‘tanh’ (20). The solver covers three training algorithms: ADAM, LBFGS, and SGD. The maximum number of iterations (max_iter) sets the upper bound on the number of training epochs.

(17) Identity: (f(x)=x)

(18) RELU: (f(x)=max(0,x))

(19) Sigmoid: (f(x)=1/(1+exp(−x)))

(20) $$\mathrm{Tan}h:\;(f(x)=\frac{e^{x}-e^{-x}}{e^{x}+e^{-x}})$$

## 5. Training Results and Evaluation

Three machine learning models (SVR, RF, and ANN) were developed based on the RBF interpolation-augmented dataset to predict the printing density of IN718 nickel-based superalloy. The study first compares the prediction accuracy of each ML model on the training set. Subsequently, the performance of the different models is evaluated by comparing their prediction results with the original data.

### 5.1. Machine Learning Model Parameter Configuration

In this study, the hyperparameters of the SVR model were optimized, selecting the Radial Basis Function (RBF) as the kernel function (21):

(21) $$K(x_{i},x_{j})=exp(-\gamma {\left\|x_{i}{-x}_{j}\right\|}^{2})$$

where γ is the kernel function parameter of the SVR model.

The optimal hyperparameter configuration for the SVR model is {C = 150, ε = 0.001, γ = 1}. The optimal configuration for the Random Forest model is {max_depth: 20, min_samples_leaf: 1, min_samples_split: 2, n_estimators: 200}. The optimal configuration for the ANN model is {activation: ‘tanh’, hidden_layer_sizes: (50, 40, 20), max_iter: 1000, solver: ‘lbfgs’}. The results of all configurations are listed in Table 6.

### 5.2. Test Set Fitting Performance of Machine Learning Models

The test set performance and corresponding R^2^ results are listed in Table 7. Based on the figures showing validation-set prediction results and R^2^ values, the optimized model’s performance can be compared. Among them, the ANN model (average R^2^ = 0.9269$) outperformed the RF model (average R^2^ = 0.8393$) and the SVR model (average R^2^ = 0.9148$).

**Figure 16:**
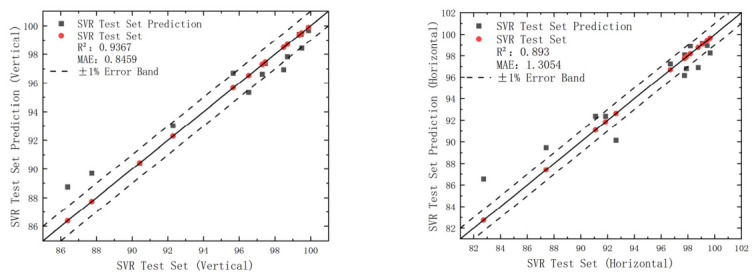
SVR Test Set Results.

**Figure 17:**
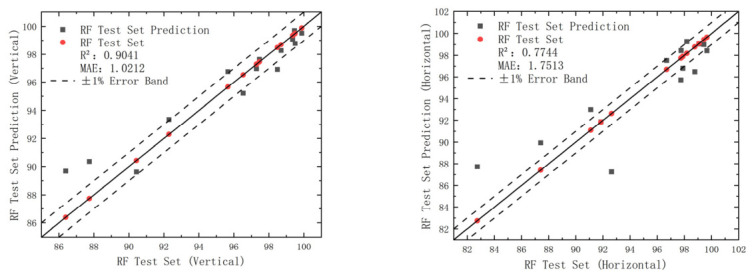
RF Test Set Results.

**Figure 18:**
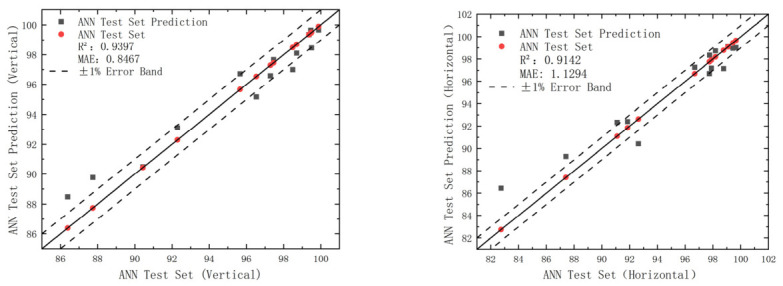
ANN Test Set Results.

**Figure 19:**
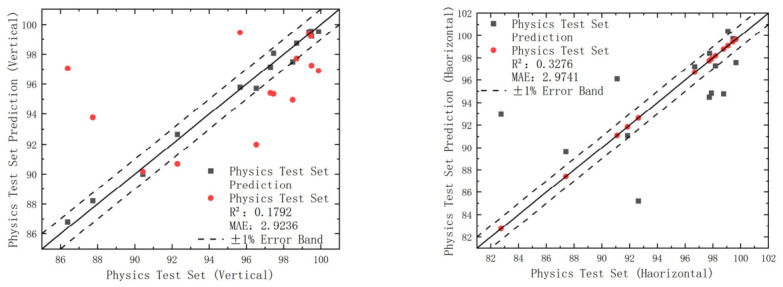
Physics Test Set Results.

The density results obtained after adjusting the hyperparameters and parameters of the optimized machine learning models are compared with the test set results. The SVR test set results are shown in Figure 16, the RF test set results in Figure 17, and the ANN test set results in Figure 18. The test set for the E_vol quadratic fitting is shown in Figure 19.

In Leave-One-Out Cross-Validation (LOO-CV), the prediction accuracy of all three machine learning models for relative density significantly outperforms the physical baseline of quadratic fitting based on volumetric energy density (E_vol) (average R^2^ = 0.2534). Among them, the Artificial Neural Network (ANN) exhibits the best predictive performance, with an average R^2^ of 0.9269 and Mean Absolute Errors (MAEs) of 0.8467 and 1.1294 in the vertical and horizontal directions, respectively. Support Vector Regression (SVR) performs second best (average R^2^ = 0.9148), while Random Forest (RF), as a representative ensemble learning model, shows moderate performance (average R^2^ = 0.8393) due to generalization bottlenecks constrained by the small sample size and tree-based structure. This indicates that non-linear machine learning models can accurately capture the complex coupling relationships of multi-process parameters in additive manufacturing.

Although E_vol integrates the effects of four process parameters—laser power (P), scan speed (v), hatch spacing (h), and layer thickness—the prediction accuracy of the quadratic fitting model with E_vol as the single independent variable is extremely limited. The fundamental reason is that the same E_vol value can be achieved by different combinations of P, v, and h. These varying combinations exert significantly different impacts on melt pool dynamics, thermal gradient distribution, and solidification behavior, resulting in a large dispersion of measured density under the same E_vol [21]. Consequently, single-parameter physical formulas cannot effectively distinguish such differences. Therefore, it is more appropriate to use deep learning algorithms for regression training.

### 5.3. Ablation Study

To quantitatively evaluate the driving effect of physics-derived features on the model’s predictive performance, this study designs an ablation study using the Random Forest model as the vehicle. The input feature set (S1–S4) is incrementally expanded according to the hierarchical physical significance of the features, and the LOO-CV framework is used to evaluate the prediction accuracy of each scheme. The design schemes are as follows: (Table 8).

Consistent with the main model, the LOO-CV framework is adopted, using the coefficient of determination (R^2^) as the evaluation metric, encompassing both the vertical and horizontal directions:

(22) $${\bar{R}}^{2}=\frac{R_{vertical}^{2}+R_{horizontal}^{2}}{2}$$

The four resulting sets of ${\bar{R}}^{2}$ are as follows: (Table 9).

The results indicate that the baseline scheme S1, which uses the original three-dimensional parameters (P, v, h) as input, yielded an average ${\bar{R}}^{2}$ of 0.8147, demonstrating that the primary process parameters already possess a certain level of predictive capability. After introducing the volumetric energy density E_vol (S2), the average ${\bar{R}}^{2}$ increased to 0.8341, an improvement of 0.0194. This suggests that the explicit physical mapping of E_vol to density effectively compensates for the model’s limitations in implicitly learning energy coupling relationships from limited samples. Upon further introducing the linear energy density E_line and $h^{2}$ (S3), the performance remained largely stable (${\bar{R}}^{2}$ = 0.8343), and after incorporating the complete 8-dimensional feature set (S4), the average ${\bar{R}}^{2}$ reached 0.8393. These results demonstrate that E_vol is the single physical feature that makes the greatest contribution and that the full 8-dimensional feature set further enhances the model’s overall generalization capability.

### 5.4. Sensitivity Analysis

#### Feature Importance

Based on the trained final Random Forest model, the Mean Decrease in Impurity (MDI)-based feature importance method is employed to quantitatively evaluate the contribution weight of each input feature to the model’s predictive capability, as shown in the following equation:

(23) $${FI}_{j}=\frac{1}{T}\sum_{t=1}^{T}\sum_{s\in S_{t}:v(s)=j}p(s)\cdot \Delta I(s)$$

where T is the number of decision trees; $S_{t}$ is the set of all nodes in the t-th tree; v(s) = j indicates that node s is split using feature j; p(s) is the proportion of samples reaching node s; and ΔI(s) is the reduction in impurity resulting from the split at that node.

Normalize the values such that the sum of all feature importances equals 1:

(24) $$\sum_{j=1}^{8}{FI}_{j}=1$$

Considering both the vertical and horizontal directions:

(25) $${\bar{FI}}_{j}=\frac{{FI}_{j}^{vertical}+{FI}_{j}^{horizontal}}{2}$$

**Figure 20:**
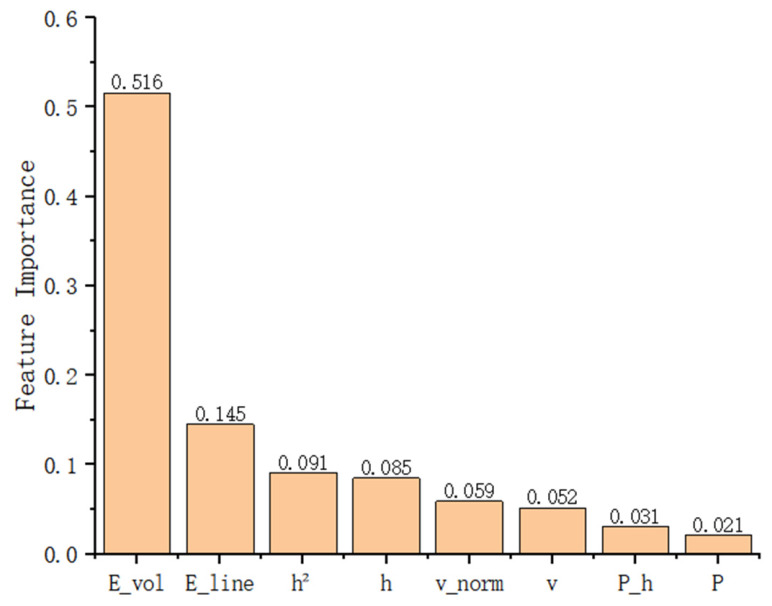
Ranking of sensitivity analysis results.

The results are shown in Figure 20.

The analysis results indicate that the average feature importance of volumetric energy density (E_vol) is 0.516, far surpassing that of all other features. This aligns closely with its physical significance: E_vol directly quantifies the laser’s thermal input into the powder and serves as the core physical quantity that determines the completeness of powder melting. Linear energy density (E_line, FI = 0.145) and the square of the hatch spacing ($h^{2}$, FI = 0.091) rank second and third, respectively, reflecting the sufficiency of melt track formation. It is worth noting that the importance of the raw process parameter P is only 0.021, indicating that the influence of laser power on density is manifested primarily through the composite metric E_vol, rather than acting independently. These results validate the rationale for introducing physics-derived features in this study: compared to raw parameters, physical features more directly reflect the SLM-forming mechanism, thereby indirectly enhancing the model’s interpretability.

### 5.5. Analysis of PDPs for Five Input Features

To further elucidate the predictive mechanism of the optimal machine learning model (ANN), this study employs Partial Dependence Plots (PDP) [28] to visualize the marginal effects of each input feature on the predicted density. PDP can reflect the non-linear impact of a single feature on the model output while keeping other features constant; it is currently a widely used model diagnostic tool in the field of Explainable Machine Learning (Explainable ML).

For the i-th feature, the PDP is calculated as follows: on a uniformly distributed sequence of grid values, the feature is fixed to these grid values one by one, while the remaining features are kept at their true values from the original 14 sets of experimental data. The marginal prediction mean curve is then obtained by averaging the Individual Conditional Expectation (ICE) curves.

(26) $${ICE}^{\left(i\right)}(x_{j})=\hat{f}\left(x_{j},x_{C}^{\left(i\right)}\right),\;i=1,2,\ldots ,n$$

(27) $${\hat{f}}_{x_{j}}(x_{j})=\frac{1}{n}\sum_{i=1}^{n}{ICE}^{\left(i\right)}(x_{j})$$

Each ICE curve represents the influence of changes in the target feature on the prediction when the remaining feature values of the i-th sample are fixed. The PDP is the point-wise average of all ICE curves [29].

In this study, the background dataset for ICE calculation utilizes only the original 14 sets of experimental data (rather than synthetic samples after data augmentation) to ensure that the PDP curves reflect the actual physical experimental distribution and to avoid interpolation bias introduced by synthetic data. Vertical density and horizontal density are predicted by two independently trained ANN models; therefore, each corresponds to a separate PDP curve.

**Figure 21:**
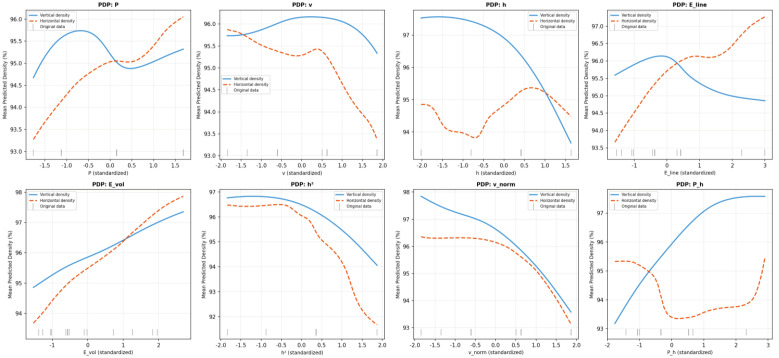
PDP analysis results.

Figure 21 displays the PDP analysis results for the 8 input features, where the solid blue line represents vertical density, the dashed orange line represents horizontal density, the ordinate (*y*-axis) is the mean predicted density (%), and the abscissa (*x*-axis) represents the standardized feature values.

The vertical and horizontal density curves for volumetric energy density (E_vol) both exhibit a clear monotonically increasing trend, indicating that density in both directions increases significantly as it rises. This is consistent with the energy input mechanism in the SLM-forming process: the higher the laser energy per unit volume, the more fully the powder melts, resulting in reduced porosity and increased material density [2,20]. Among all features, the PDP result for this parameter shows the highest degree of agreement with physical laws, and the response trends in both directions are basically consistent.

The PDP curves for both scanning speed (v) and normalized speed (V_norm) exhibit a negative correlation, meaning that the predicted density gradually decreases as scanning speed increases [2,5]. This is primarily because an increase in scanning speed leads to a reduction in laser energy input per unit length, thereby lowering melting adequacy. As a normalized form of scanning speed, V_{norm} = v/1000 maintains a consistent trend with the original variable v, further verifying the physical rationality of the feature transformation process.

Regarding scanning spacing (h) and squared spacing (h^2), the PDP curve for scanning spacing h shows that vertical density decreases significantly as spacing increases, indicating that larger scanning spacing reduces the overlap rate between melt tracks, thereby leading to increased porosity defects, which is consistent with SLM process principles [2,4]. In contrast, the PDP curve for the non-linear feature h^2 shows a more significant downward trend, indicating that the impact of scanning spacing on density has a certain non-linear enhancement characteristic.

The vertical density curve for laser power (P) exhibits some local fluctuations in the low-to-medium power range, but it still shows an overall upward trend; horizontal density shows a relatively stable monotonically increasing relationship as power increases. Overall, this result is consistent with the physical understanding that “higher laser power leads to more sufficient melt pool melting” [2].

For the two derived features, line energy density (E_line) and power-spacing ratio (P/h), the PDP curves for both directions exhibit certain differences. Specifically, E_{line} = P/v and P/h are essentially combination variables between laser power and process parameters, and they have strong multicollinearity with the basic features P, v, and h. Under small-sample conditions, the two independently trained ANN models learned different local marginal effects for these derived features. This difference may reflect the anisotropy of the melt pool solidification behavior during the SLM-forming process, where the difference in the laser scanning direction relative to the tensile direction leads to differences in the densification formation mechanism [6,7].

### 5.6. Validation Set Performance and Discussion

**Figure 22:**
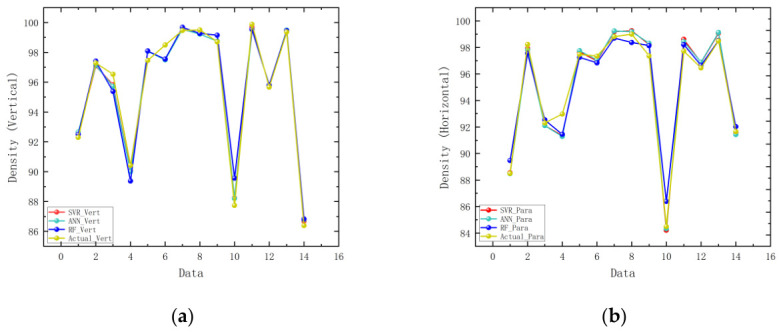
Comparison Between the Predicted Density of the RBF-Based Models and the Experimental Data.

The prediction accuracy of three different machine learning models under the RBF augmentation strategy was compared. Finally, the optimized models were used to predict Density in both parallel and vertical directions on the test set (original dataset), and the generalization capability of the models was evaluated. Figure 22 compares the predictions of different models across various test sets, with the corresponding R^2^ and MAE values listed in Table 7.

## 6. Conclusions

To address the challenge of modeling the complex non-linear mapping between process parameters and density in the Selective Laser Melting (SLM) of IN718 nickel-based alloy, this study proposes an intelligent density prediction framework that integrates physics-constrained data augmentation, multi-dimensional physical feature engineering, systematic grid search hyperparameter optimization, and model interpretability analysis via Partial Dependence Plots (PDP). In industrial production, SLM process parameter optimization typically relies on extensive trial-and-error experiments, which are characterized by long cycles and high costs. This framework requires only 14 original experimental datasets and, through physics-constrained enhancement and machine learning modeling, enables accurate prediction of density in both vertical and horizontal directions within a process window of P in [200, 335] W, v in [600, 1200] mm/s, and h in [0.06, 0.12] mm (average ANN R^2^ = 0.9269, MAE < 1.2%). This approach can reduce the cost of process parameter screening by over 60%, providing an efficient data-driven support tool for the rapid determination of process windows for IN718 aerospace components. Through systematic multi-strategy comparative analysis and interpretability verification, the following main conclusions are drawn:

(1) Using laser power (P), scanning speed (v), and hatch spacing (h) as process variables, a raw experimental dataset was collected through 14 systematically designed SLM printing experiments, containing density measurements in both vertical and horizontal directions. To address the overfitting that easily arises in direct modeling under small-sample conditions, three data augmentation strategies were systematically compared: RBF thin-plate spline interpolation, Generative Adversarial Networks (GANs), and physics-inspired augmentation with volumetric energy density constraints (RBF + E).

(2) During the feature engineering stage, eight physically correlated features were introduced, including volumetric energy density (E_vol) and linear energy density (E_line), to enhance the model’s generalization capability. The Pearson Correlation Coefficient (PCC) was used to comparatively analyze the correlation structure between the original and augmented data. The high consistency between the RBF-interpolated augmented data and the original data in statistical patterns was verified, confirming its validity and usability. Volumetric energy density showed the strongest positive correlation with density (PCC = 0.52), indicating it is the primary physical parameter governing SLM-forming quality.

(3) To address the challenge of model selection under small-sample conditions, all three machine learning models (SVR, RF, and ANN) were subjected to systematic hyperparameter optimization via exhaustive grid search combined with LOO-CV. For SVR, the optimal configuration was determined as {C = 150, ε = 0.001, γ = RBF kernel}; for RF, the best combination of n_estimators, max_depth, min_samples_split, and min_samples_leaf was identified; and for ANN, the optimal hidden layer architecture, activation function, and solver were selected. Experimental results demonstrate that the RBF augmentation strategy combined with the SVR-RBF model achieves the best overall performance, with a test set vertical Density R^2^ of 0.9908 and a Mean Absolute Error (MAE) of only 0.3347%, significantly outperforming other model combinations under GAN augmentation and physics-inspired augmentation strategies. Random Forest (RF) and Artificial Neural Network (ANN) also exhibit strong predictive performance across all augmentation strategies. In summary, it is recommended to prioritize RBF interpolation for data augmentation and to combine it with the SVR-RBF model for the highest prediction accuracy. As dataset sizes increase, ANNs and RFs have greater potential for non-linear fitting and can serve as key directions for future research.

(4) To elucidate the predictive mechanism of the optimal ANN model beyond accuracy metrics, Partial Dependence Plot (PDP) analysis was conducted for all eight input features using the original 14 experimental data points as the background dataset. The PDP results confirm the physical rationality of the model: E_vol exhibits a clear monotonically increasing effect on density in both vertical and horizontal directions, consistent with the energy input mechanism governing powder melting completeness; scanning speed (v) and its normalized form (v_norm) show a negative correlation with density, reflecting reduced melting adequacy at higher speeds; hatch spacing (h) and its squared form (h^2^) exhibit a significant negative effect, confirming the role of track overlap in controlling porosity; and laser power (P) shows an overall positive trend, consistent with the understanding that higher power promotes more complete melt pool formation. The differences observed between the PDP curves for the two derived features (E_line, P/h) in the vertical and horizontal directions are attributed to the anisotropic solidification behavior of the SLM process. These findings demonstrate that the trained model has successfully learned physically meaningful process–property relationships, enhancing the interpretability and trustworthiness of the prediction framework.

## Figures and Tables

**Table 1:** Chemical Composition of IN718 Alloy (mass fraction %).

Ni	Nb	Mo	Ti	Al	Cr	C	Fe
54.00	5.29	3.11	1.01	0.38	19.72	0.03	blance

**Table 2:** Specimen Numbering and Related Printing Parameters.

No.	P (W)	v (mm/s)	h (mm)
1	200	1000	0.10
2	225	800	0.10
3	225	1000	0.10
4	225	1200	0.10
5	275	980	0.10
6	275	1000	0.10
7	335	680	0.10
8	335	1000	0.10
9	275	800	0.10
10	275	1200	0.10
11	335	600	0.10
12	275	800	0.06
13	275	800	0.08
14	275	800	0.12

**Table 3:** Average Density of Specimens.

No.	Relative Density Perpendicular to the Scan Plane (%)	Average Relative Density Perpendicular to the Scan Plane (%)	Relative Density Parallel to the Scan Plane (%)	Average Relative Density Parallel to the Scan Plane (%)
1	94.46	92.29	84.82	87.41
	92.949		91.113	
	92.421		84.82	
	89.315		88.905	
2	95.99	97.29	98.39	98.77
	97.92		99.00	
	98.94		98.94	
	98.02		98.76	
3	96.57	96.53	91.22	91.85
	98.24		90.09	
	96.63		94.44	
	94.68		91.66	
4	92.65	90.43	92.64	90.67
	87.02		87.38	
	91.7		91.93	
	90.36		90.75	
5	95.58	97.46	98.50	97.89
	97.69		97.48	
	97.56		97.26	
	99.03		98.32	
6	98.12	98.49	97.44	97.74
	99.31		97.50	
	99.33		98.20	
	97.2		97.82	
7	99.58	99.46	99.14	99.44
	99.48		99.54	
	99.13		99.44	
	99.64		99.63	
8	99.48	99.5	99.72	99.66
	99.34		99.74	
	99.46		99.76	
	99.73		99.40	
9	99.62	98.7	96.98	97.76
	97.30		97.06	
	98.39		98.99	
	99.48		97.99	
10	88.77	87.74	80.34	82.74
	87.48		82.02	
	87.15		85.51	
	87.57		83.11	
11	99.82	99.88	98.31	98.18
	99.90		97.68	
	99.88		98.31	
	99.92		98.42	
12	96.50	95.66	96.51	96.69
	95.58		97.73	
	95.97		97.05	
	94.59		95.48	
13	99.55	99.36	99.33	99.08
	99.52		97.88	
	98.97		99.32	
	99.37		99.81	
14	85.52	86.39	91.33	91.10
	89.3		89.11	
	83.1		93.21	
	87.66		90.74	

**Table 4:** Feature Definitions.

Feature Name	Formula	Physical Meaning
Laser Power	P (W)	Laser output power directly determines the thermal input to the melt pool
Scanning Speed	v (mm/s)	Laser scanning velocity affects powder heating duration
Hatch Spacing	h (mm)	Distance between adjacent scan track centers; affects track overlap ratio
Linear Energy Density	E_line = P/v	Thermal input per unit scan length; characterizes melting completeness
Volumetric Energy Density	E = P/(v·h·t)	Comprehensive energy density proxy; strongly positively correlated with Density
Squared Hatch Spacing	h^2^	Captures non-linear effects of track overlap
Normalized Speed	v_norm = v/1000	Eliminates dimensional inconsistency
Power-to-Spacing Ratio	P_h = P/(h × 1000)	Laser power density per unit hatch spacing

**Table 5:** Grid Hyperparameter Optimization Parameter Ranges.

ML Model	Tuning Entity	Range	No. of Values
SVR	K	[‘linear’, ’poly’, ’rbf’, ’sigmoid’]	4
	C	[10, 20, …, 140, 150]	15
	ε	[0.001, 0.01, 0.1, 1, 10]	5
	γ	[0.001, 0.01, 0.1, 1, 10]	5
RF	n_estimatorsn	[50, 100, 150, 200, 500]	5
	max_depth	[None, 10, 20, 30, 40, 50]	6
	min_samples_split	[2, 4, 6, 8, 10, 16]	6
	min_samples_leaf	[1, 2, 4]	3
ANN	hidden_layer_sizes.	[(*i*, *j*, *k*)] *i* ϵ [20, 30 … 70, 80] *j* ϵ [10, 20, 40] *k* ϵ [10, 15, 20]	(7, 3, 3)
	activation.	[identity, RELU, sigmoid, tanh]	4
	solver.	[adam, lbfgs, sgd]	3
	max_iter.	[100, 500, 1000, 2000, 5000]	5

**Table 6:** Optimal Hyperparameters After Optimization.

ML Model	Tuning Entity	Value
SVR	K	RBF
	C	150
	ε	0.001
	γ	1
RF	n_estimators	200
	max_depth	20
	min_samples_split	2
	min_samples_leaf	1
ANN	hidden_layer_sizes.	(50, 40, 20)
	activation.	[tanh]
	solver.	[lbfgs]
	max_iter.	1000

**Table 7:** Test Set Performance.

ML Model	R^2^ (Vertical)	R^2^ (Horizontal)	Avg. R^2^	MAE (Vertical, %)	MAE (Horizontal, %)	Avg. MAE (%)
SVR	0.9367	0.893	0.9148	0.8459	1.3054	1.0756
RF	0.9041	0.7744	0.8393	1.0212	1.7513	1.3862
ANN	0.9397	0.9142	0.9269	0.8467	1.1294	0.9881
E_vol	0.1792	0.3276	0.2534	2.9236	2.9741	2.9488

**Table 8:** The four feature combinations are as follows (S1–S4).

Dimension	Features
3	P, v, h
4	P, v, h, E_vol
6	P, v, h, E_vol, E_line, h^2^
8	P, v, h, E_vol, E_line, h^2^, v_norm, P_h

**Table 9:** The four resulting sets of ${\bar{R}}^{2}$

No.	${\bar{R}}^{2}$
S1	0.8147
S2	0.8341
S3	0.8343
S4	0.8393

## Data Availability

The original contributions presented in this study are included in the article. Further inquiries can be directed to the corresponding author.
